# Construction of an integrated human osteosarcoma database, HOsDb, based on literature mining, microarray analysis, and database retrieval

**DOI:** 10.1186/s12885-020-06719-2

**Published:** 2020-05-06

**Authors:** Yifu Sun, Lishan Wang, Changkuan Li, Rui Gu, Weidong Zang, Wei Song, Peng Xia

**Affiliations:** 1 Department of Orthopedics, China-Japan Union Hospital of Jilin University, Changchun, 130033 P.R. China; 2 Eryun (Shanghai) Information Technology Co., Ltd, Shanghai, 200241 P.R. China; 3 Department of Orthopedics, The Second Hospital of Jilin University, No.218 Ziqiang Street, Changchun, 130022 China

**Keywords:** Osteosarcoma, HOsDb, www.hosdatabase.com

## Abstract

**Background:**

Osteosarcoma (OS) is the most frequent primary malignancy of bone with a high incidence in adolescence. This study aimed to construct a publicly available, integrated database of human OS, named HOsDb.

**Methods:**

Microarray data, current databases, and a literature search of PubMed were used to extract information relevant to human OS-related genes and their transcription factors (TFs) and single nucleotide polymorphisms (SNPs), as well as methylation sites and microRNAs (miRNAs). This information was collated for constructing the HOsDb.

**Results:**

In total, we identified 7191 OS tumor-related genes, 763 OS metastasis-related genes, and 1589 OS drug-related genes, corresponding to 190,362, 21,131, and 41,135 gene-TF pairs, respectively, 3,749,490, 358,361, and 767,674 gene-miRNA pairs, respectively; and 28,386, 2532, and 3943 SNPs, respectively. Additionally, 240 OS-related miRNAs, 1695 genes with copy number variations in OS, and 18 genes with methylation sites in OS were identified. These data were collated to construct the HOsDb, which is available at www.hosdatabase.com. Users can search OS-related molecules using this database.

**Conclusion:**

The HOsDb provides a platform that is comprehensive, quick, and easily accessible, and it will enrich our current knowledge of OS.

## Background

Osteosarcoma (OS), the most frequent primary malignancy of bone, commonly occurs in the metaphyseal region of the long bones, developing at sites of rapid bone growth [1]. OS commonly affects children, adolescents, and young adults. The annual incidence of OS in the general population is 2–3/million/year, while in adolescence, especially from 15 to 19 years of age, OS incidence reaches 8–11/million/year [2]. OS accounts for 15% of all solid extracranial cancers in people aged 15–19 years [3]. OS can be divided into several subtypes, such as osteoblastic, chondroblastic, fibroblastic, small cell, telangiectatic, high-grade surface, extra-skeletal, and other lower-grade forms, including periosteal and parosteal [4]. Some OS cases are likely to have a genetic basis, and numerous hereditary disorders associated with germline alterations of tumor suppressor genes have been found in patients with OS, such as hereditary retinoblastoma [5] and Li-Fraumeni cancer family syndrome [6, 7]. However, the mechanisms underlying the pathogenesis of OS remain largely unclear.

Many databases have been developed to investigate the association between certain molecules of interest and disease pathogenesis from different perspectives. For instance, Online Mendelian Inheritance in Man (OMIM) [8] contains information on the relationship between the phenotype and genotype of all known Mendelian disorders. Wikigenes [9] is a portal that provides information about genes, proteins, chemical compounds and their reported associations with various diseases. The miR2Disease [10] and Human microRNA Disease Database (HMDD) [11] aim to provide comprehensive collection of microRNAs (miRNAs) associated with various human diseases. MethyCancer [12] contains highly integrated data regarding cancer-related genes, DNA methylation sites, and information on cancer from public resources. TRANSFAC is a database of transcription factors (TFs), which offers an integrated system for predicting gene expression regulation [13]. Although research data regarding OS has accumulated during the past decades, to the best of our knowledge, there is only one available database specifically focusing on OS molecular biology, called Osteosarcoma Database [14]. Nevertheless, only 911 OS-associated genes and 81 miRNAs collected through manual literature mining are included in this database, and there is no information available regarding other OS-related molecules, such as TFs or methylation sites [14]. The development of high-throughput laboratory techniques, such as microarray analysis, has enabled generation of large quantities of data associated with OS, which are an important resource for exploration of potential OS-related molecules, including genes, miRNAs, and copy number variations (CNVs) [15–18]. While these data provide insight into certain aspects of OS, they are not assembled together in a structured format. Thus, there is a need to establish an integrated, OS-specific database or platform of OS-related genes, TFs, methylation sites, and miRNAs.

We collected detailed OS-related data, including OS-related genes, TFs, single nucleotide polymorphisms (SNPs), miRNAs, methylation sites, and CNVs by analyzing several microarray deposits in the Gene Expression Omnibus (GEO) data repository, searching current databases, and mining the literature in PubMed. Using these data, we aimed to construct a publicly available, integrated database of human OS to facilitate the exploration of human OS-related molecules and create a unique resource for research into this disease.

### Construction and content

#### Database construction

**Fig. 1:**
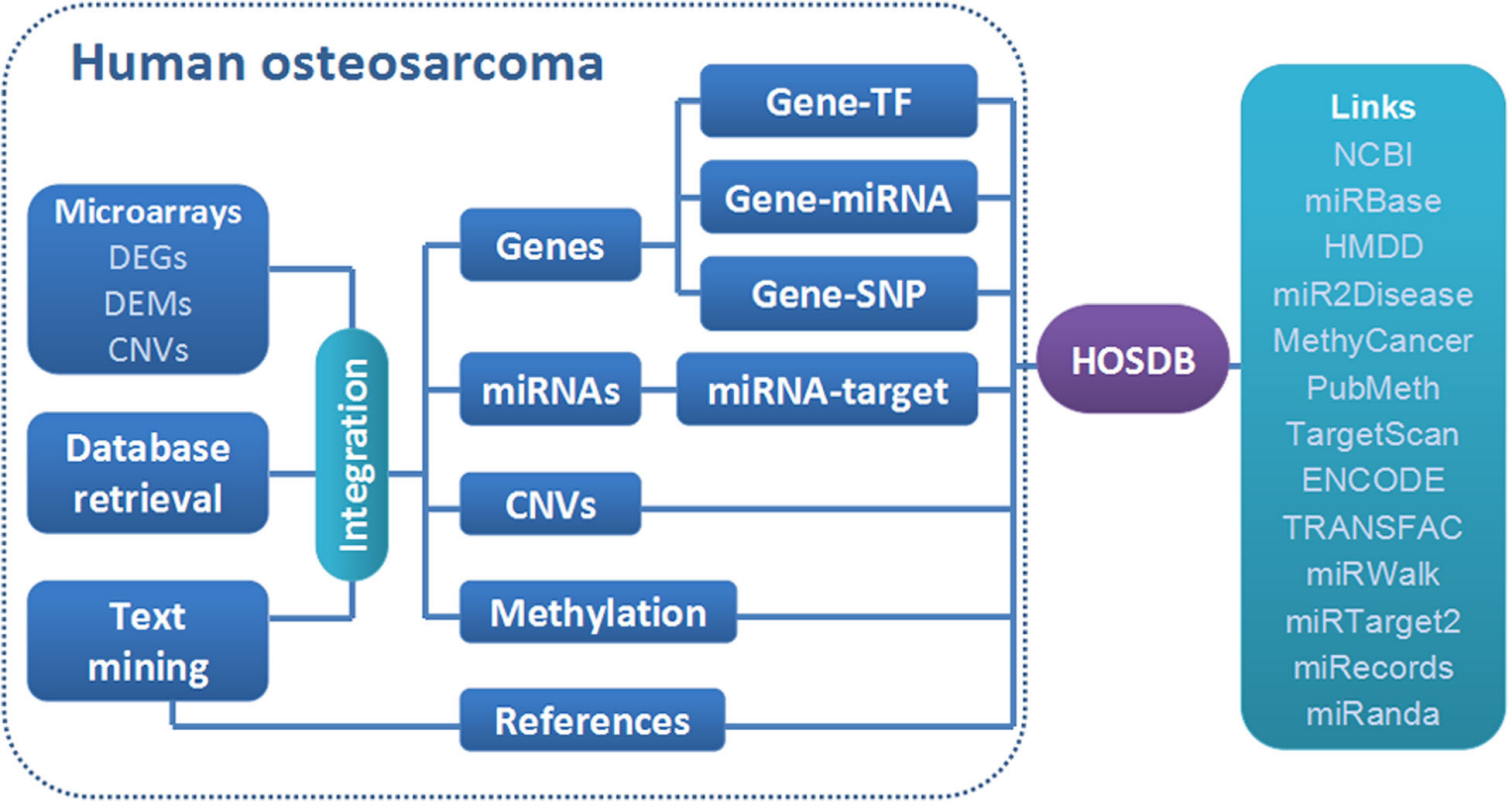
Construction of the HOsDb HOsDb: human osteosarcoma database; DEGs: differentially expressed genes; DEMs: differentially expressed miRNAs; CNVs: copy number variations; miRNA: microRNAs; TFs: transcription factors; SNPs: single nucleotide polymorphisms

The integrated database of human OS, named HOsDb, aims to provide a high-quality collection of human OS-related genes, methylation sites, CNVs, miRNAs, TFs, and SNPs based on literature mining, microarray analysis, and database retrieval. The data collection and processing steps are illustrated in Fig. 1.

#### OS-related genes

Initially, mRNA expression microarrays related to OS were downloaded from the GEO database [19]. Detailed information regarding the datasets used, such as the GEO accession number and sample type and size, is shown in Table 1. The corresponding experimental conditions were tumor vs. normal, metastasis vs. non-metastasis, or drug-treated vs. untreated. Raw Affymetrix data in CEL file format were read using Affy [20] and normalized using the robust microarray analysis (RMA) method [21]. The downloaded normalized expression matrix was used for analysis of data generated using Illumina and Agilent platforms. Differentially expressed genes (DEGs), defined as OS-related DEGs, were identified using the Linear Models for Microarray and RNA-Seq Data (limma) package [22] with a cut-off value of |log fold change (FC)| > 1 and false discovery rate (FDR) [23] < 0.05. A total of 6964 OS tumor-related, 685 OS metastasis-related, and 1589 OS drug-related DEGs were identified (Table 2). Literature mining of the PubMed collection was used to generate a list of known OS tumor-related and OS metastasis-related genes. A total of 505 genes related to OS tumor and 87 genes related to OS metastasis were found in the published literature. A list of OS-related genes was then collated by integrating OS-related DEGs identified by microarray analysis and OS-related genes identified by literature mining. Using this approach, 7191 OS tumor-related genes (Supplementary Table 1), 763 OS metastasis-related genes, and 1589 OS drug-related genes were identified (Table 2).

**Table 1:** Information of the included datasets

Category		GEO accession number	Platform	Sample type	Sample size	Experiment design
Gene	Tumor vs. normal	GSE11414	GPL6244	Cell lines	Tumor: 4; normal: 2	genome-wide comparison of gene expression and identified genes that are differentialy expressed in osteosarcoma (U2OS, MG63) cell lines relative to normal human osteoblasts (HOB)
		GSE12865	GPL6244	Tumor tissues and normal cell line	Tumor: 12; normal: 2	genome-wide comparison of gene expression and identified genes that are differentialy expressed in osteosarcoma tumour samples relative to normal human osteoblasts (HOB)
		GSE14359	GPL96	Tumor tissues and normal cell line	Tumor: 18; normal: 2	mRNA from 5 frozen conventional osteosarcoma and 4 osteosarcoma lung metastases tumor samples and mRNA from fresh primary osteoblast cells (HOB) were extracted and hybridized to HG U133A microarrays
		GSE16088	GPL96	Tumor and normal tissues, as well as tumor cell lines	Tumor: 17; normal: 6	Profiles of human osteosarcoma and three normal tissues, single channel design
		GSE19276	GPL6848	Tumor and normal tissues	Tumor: 44; normal: 5	Gene expression profiling of primary osteosarcoma biopsies and compared the results to gene expression profiling of non-malignant bone to identify differentially expressed genes unique to OS in the context of the bone micorenvironment
		GSE28424	GPL13376	Tumor cell lines and normal tissues	Tumor: 19; normal: 4	19 osteosarcoma cell lines, 4 normal bones used as controls. No replicates. The group of osteosarcomas are compared to the group of normal bones.
		GSE30807	GPL570	Tumor cell lines and normal bone mesenchymal stem cells	Tumor: 2; normal: 1	To analysis stem/progenitor cell-associated genes and molecules involved in regulation of self-renewal signaling pathways of cancer stem cells between UT2 cells and its parent cells: U2OS (MSC works as positive control here)
		GSE36001	GPL6102	Tumor cell lines and normal osteoblast and bone cells.	Tumor: 19; normal: 6	Comparison of gene expression patterns in 19 osteosarcoma cell lines and 6 normal samples (osteoblasts and bones)
		GSE42352	GPL10295	Tumor cell lines, pre-chemotherapy biopsies, osteoblasts, mesenchymal stem cells	Tumor: 103; normal: 15	Gene set analysis on previously published genome-wide gene expression data of osteosarcoma cell lines (*n* = 19) and osteosarcoma pre-chemotherapy biopsies (*n* = 84), and characterizing expression of the insulin-like growth factor receptor signaling pathways in human osteosarcoma as compared with osteoblasts and with the hypothesized progenitor cells of osteosarcoma - mesenchymal stem cells.
		GSE56001	GPL10558	Tumor cells and normal mesenchymal stem cells	Tumor: 3; normal: 9	Analysis of gene changes in different genes modulation in mesenchymal stem cells and compared to primary human osteosarcoma cells
		GSE9508	GPL6076	Tumor and normal biopsies	Tumor: 34; normal: 5	Two-colour experiment. 7 samples for non-metastatic patients, 6 of which are analyzed in duplicate (dye-swaps); 11 samples for metastatic patients, 10 of which are analyzed in duplicate (dye-swaps); 5 samples of non-malignant bone analyzed individualy, no dye-swaps (i.e. 5 biological replicates).
	Metastasis vs. non-metastasis	GSE14359	GPL96	Conventional osteosarcoma and lung metastases tumor samples	Metastasis: 8; non-metastasis: 10	mRNA from 5 frozen conventional osteosarcoma and 4 osteosarcoma lung metastases tumor samples and mRNA from fresh primary osteoblast cells (HOB) were extracted and hybridized to HG U133A microarrays
		GSE18947	GPL570	Low and high metastatic potential cell sublines	Low metastasis: 3; high metastasis: 3	The assay was performed among three pairs of cublines, the first two pairs of sublines comes from the different passage of sublines established with orthotopic transplantation under the established cell line named Sosp-9607, the other pair was screened by the tail-vein injection method of commercial avaliable cell line-Saos-2.
		GSE21257	GPL10295	Metastatic and non-metastatic tumor biopsies	Metastasis: 34; non-metastasis: 19	Pre-chemotherapy biopsies of osteosarcoma patients who developed metastases within 5 yrs. (*n* = 34) were compared with pre-chemotherapy biopsies of osteosarcoma patients who did not develop metastases within 5 yrs. (n = 19)
		GSE9508	GPL6076	Metastatic and non-metastatic tumor biopsies	Metastasis: 21; non-metastasis: 13	Two-colour experiment. 7 samples for non-metastatic patients, 6 of which are analyzed in duplicate (dye-swaps); 11 samples for metastatic patients, 10 of which are analyzed in duplicate (dye-swaps); 5 samples of non-malignant bone analyzed individualy, no dye-swaps (i.e. 5 biological replicates).
	Drug-treated vs. untreated	GSE16089	GPL570	Methotrexate-sensitive and –resistant Saos-2 cells	Methotrexate-sensitive samples: 3; methotrexate-resistant samples: 3	Two cell lines are compared, which are Saos-2 osteosarcoma cells sensitive to methotrexate and Saos-2 cells resistant to 10e-6 M methotrexate. Six samples are provided which correspond to triplicates of each cell line.
		GSE24401	GPL1456	Atorvastatin-treated and -untreated Saos-2 cells	Atorvastatin-treated samples: 3; atorvastatin-untreated samples: 3	Dye balance-experiment comparing atorvastatin treated Saos-2 cells versus untreated cells at 6, 15 and 24 h using 2 biological replicates
miRNA		GSE28423	GPL8227	Tumor cell lines and normal bones	Tumor cell lines: 19; normal bones: 4	19 osteosarcoma cell lines, 4 normal bones used as controls. No replicates. The group of osteosarcomas are compared to the group of normal bones.
CNV		GSE12830	GPL4091, GPL9128	Tumor tissues	20	Integrative whole-genome analysis of DNA copy number, promoter methylation and gene expression using 10 osteosarcomas with 2 biological replicates
		GSE7077	GPL2879	Four osteosarcoma-derived cell lines: U-2 OS, HOS, MG-63 and SAOS-2	4	To utilize oligonucleotide array CGH to identify microaberrations in osteosarcomas, likely to contain genes involved in osteosarcoma tumor oncogenesis. A better understanding of the underlying molecular genetic events leading to tumor initiation and progression could result in the identification of prognostic markers and therapeutic targets.
		GSE9654	GPL2879		10	To utilize oligonucleotide array CGH and FISH analysis to derive possible genomic signatures of chromosomal instability in osteosarcoma tumors

“-” in the column of “PubMed ID” means that there is no published study so far. *GEO* Gene Expression Omnibus, *CGH* Comparative genomic hybridization, *FISH* Fluorescence in situ hybridization

**Table 2:** Results of data collection and analysis

	Tumor vs. normal	Metastasis vs. non-metastasis	Drug-treated vs. untreated
**OS-related gene**			
DEG (mRNA expression microarray)	6964	685	1589
Known gene (text mining)	505	87	–
Total OS-related gene	7191	763	1589
Gene-TF pair (database)	190,362	21,131	41,135
Gene-miRNA pair (database)	3,749,490	358,361	767,674
Gene-SNP (database)	28,386	2532	3943
**OS-related miRNA**			
DEM (miRNA expression microarray)	209	–	–
miRNA (database)	31	–	–
Total OS-related miRNA	240		
**OS-related CNV**			
CNV (CGH microarray)	1695	–	–
**OS-related methylation**			
Gene methylation (database)	18	–	–

*OS* Osteosarcoma, *DEG* Differentially expressed gene, *DEM* Differentially expressed miRNA, *miRNA* microRNA, *CNV* Copy number variation, *CGH* Comparative genomic hybridization

A list of TFs targeting OS-related genes was obtained from the TRANSFAC [24] and ENCODE databases [25]. We found 299 OS tumor-, 207 OS metastasis-, and 194 OS drug-related TFs, which corresponded to 190,362, 21,131, and 41,135 gene-TF pairs, respectively (Table 2). The miRNAs targeting OS-related genes were extracted from existing databases, including miRanda (Good mirSVR score part; release: August 2010) [26], miRecords (version 4) [27], miRTarget2 (version 4) [28], miRWalk (validated targets only) [29], and TargetScan (release 6.2) [30]. A total of 3,749,490, 358,361, and 767,674 gene-miRNA pairs related to OS tumor, metastasis, and drug treatments, respectively, were identified (Table 2). SNPs in OS-related genes were extracted from the National Center for Biotechnology Information (NCBI) dbSNP database (updated on 2014.05.29) [31]. We found 28,386, 2532, and 3943 SNPs in genes related to OS tumor, metastasis, and drug treatment, respectively (Table 2).

#### OS-related miRNAs

Normalized miRNA expression microarray data related to OS were also downloaded from the GEO database (Table 1). Differentially expressed miRNAs (DEMs) were identified using the limma package with a cutoff value of |logFC| > 1 and FDR < 0.05. Known OS-related miRNAs were extracted from the miR2Disease database (updated on 2011.04.14) [10] and HMDD database (updated on 2012.09.09) [11]. In total, 209 OS-related DEMs were identified based on miRNA expression microarray, and 31 known OS-related miRNAs were identified in the miR2Disease and HMDD databases, generating a final count of 240 OS-related miRNAs for inclusion (Table 2).

#### OS-related CNVs

Normalized, comparative genomic hybridization (CGH) microarray data were downloaded from the GEO database (Table 1) and analyzed using DNAcopy [32] and cghMCR packages [33]. The criteria were set at (Segment Gain or Loss (> 0.2 and incidence > 30%. A total of 1695 genes with CNVs in OS were identified (Table 2).

#### OS-related methylation sites

MethyCancer [12] and PubMeth [34] databases were searched using the keyword “osteosarcoma.” Eighteen genes with methylation sites related to OS were identified for further analysis (Table 2).

#### Data storage

The data obtained using the methods described were collated and used to construct the integrated human OS database (HOsDb), which is available for use at www.hosdatabase.com. HOsDb is a one-stop comprehensive platform for OS researchers.

## Database description

**Fig. 2:**
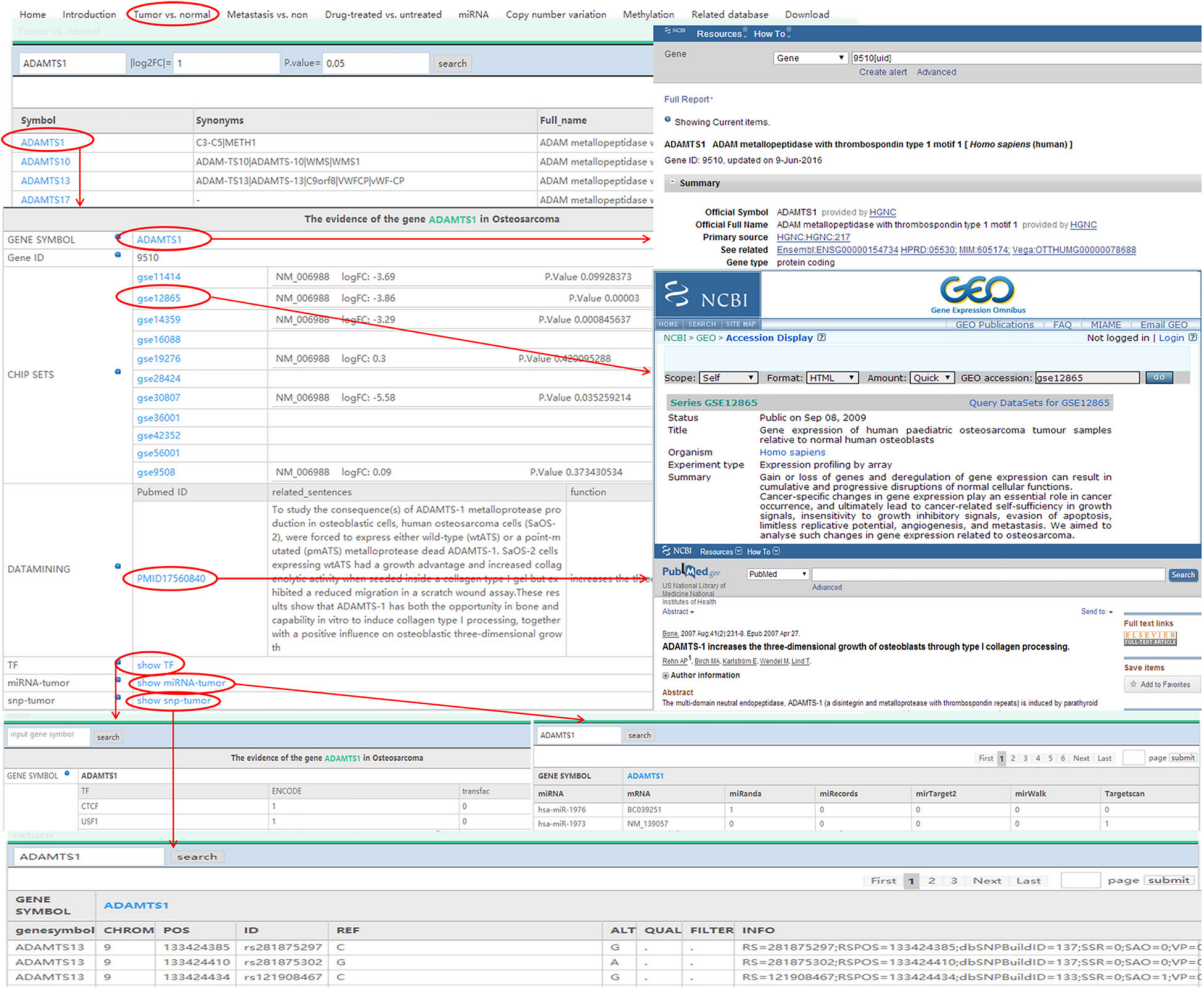
Schematic diagram of the workflow for collating OS-related genes OS: osteosarcoma; HOsDb: human osteosarcoma database; TF: transcription factor; miRNA: microRNA; SNP: single nucleotide polymorphism; ID: identifier. ‘Tumor vs. normal,’ ‘Metastasis vs. non,’ and ‘Drug-treated vs. untreated’ sections on the homepage are all focused on OS-related genes

**Fig. 3:**
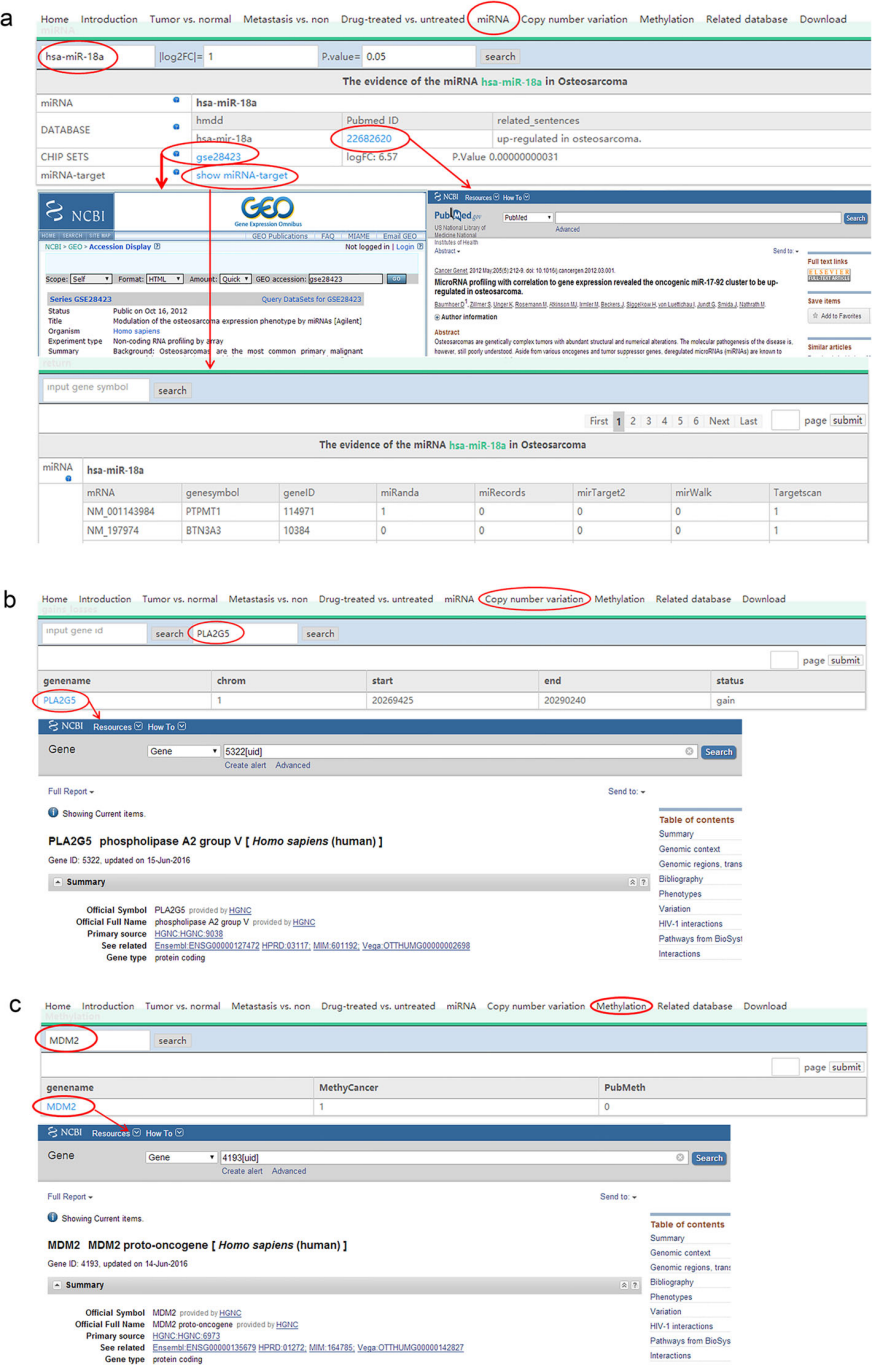
Schematic diagram of the workflow for collating OS-related miRNAs, CNVs, and methylation sitesa) miRNAs b) CNVs c) methylation sites. OS: osteosarcoma; HOsDb: human osteosarcoma database; miRNAs: microRNAs; CNV: copy number variation; ID: identifier

The HOsDb is a search engine that can be used to search detailed information on each OS-related term stored in the database. Terms include ‘Home,’ ‘Introduction,’ ‘Tumor vs. normal,’ ‘Metastasis vs. non,’ ‘Drug-treated vs. untreated,’ ‘miRNA,’ ‘copy number variation,’ ‘methylation,’ ‘Related database,’ and ‘Download.’ The ‘Tumor vs. normal,’ ‘Metastasis vs. non,’ and ‘Drug-treated vs untreated’ terms on the home page focus on OS-related genes, as well as TFs, miRNAs and SNPs targeting OS-related genes. Users can query a gene symbol in the search bar located at the top of the linked pages. After inputting the gene symbol, all information related to that gene will be displayed in a new page, including gene/TF/miRNA/SNP symbol, synonyms, full name, logFC, *p*-value, GEO microarray ID, gene/miRNA regulation direction in OS, miRNA targets, and links to publications in PubMed. To see more details about their gene of interest, users can click on the gene symbol link, and the NCBI page and results related to the gene of interest will appear (Fig. 2). The ‘miRNA’ term links users to a list of OS-related miRNAs, and users can search a particular miRNA by inputting its symbol in the search bar. Notably, users can define their own thresholds (logFC and p-value) for gene or miRNA expression. However, the default settings are logFC > 1 and p-value < 0.05 (Fig. 3a). The ‘copy number variation’ term generates a list of genes with CNVs in OS. Users can query whether a certain gene undergoes changes in copy number in OS or not by inputting the corresponding gene ID or symbol (Fig. 3b). The ‘methylation’ term lists all genes with methylation sites related to OS. Users can input a gene symbol to check whether its sequence has methylation sites in OS or not (Fig. 3c). The ‘Related database’ terms include several internal resources or databases, which are cross-linked in HOsDb, including NCBI, miRBase, HMDD, miR2Disease, MethyCancer, PubMeth, TargetScan, ENCODE, TRANSFAC, miRWalk, miRTarget2, miRecords, and miRanda. The ‘Download’ term allows users to obtain detailed information regarding DEGs, DEMs, TFs, SNPs, and CNVs that was used for HOsDb construction.

## Utility and discussion

Compared with a previously established OS database [14], the HOsDb provides more information. For example, our analyses of mRNA and miRNA expression microarrays, and CGH microarray provide a comprehensive list of candidate genes, miRNAs, and CNVs, which will assist users to navigate through the complexity of OS. Moreover, the HOsDb contains detailed gene regulation information, such as potential TF- and miRNA-gene pairs associated with OS, which is convenient for the identification of novel gene relationships involved in OS. Furthermore, information regarding SNPs in OS-related genes is provided in the HOsDb, which will help direct further studies of OS-related SNPs. The OS-related CNVs listed in the HOsDb were generated through analysis of three CGH microarray datasets. Thus, they are more reliable than those generated from a single dataset. Additionally, the HOsDb incorporates a user-friendly interface, which makes all the features easily accessible.

Although data in the HOsDb were collected using a number of different platforms and approaches, all data were normalized prior to analysis, thus adding to the reliability of our results. However, microarray data regarding OS are likely to be constantly updated in the GEO database and next-generation sequencing studies can also provide OS-related data, which will provide new insights into OS biology. This updated information will need to be added to HOsDb, once it is available. Although the HOsDb has advantages over the only other known OS-related database in its current form, we plan to update the database periodically to consistently maintain the quality of OS-related data available, and thus, keep up to date with changes and improvements in the field.

## Conclusions

The HOsDb provides a one-stop, comprehensive platform for human OS research that is quick and easily accessible. We believe that the HOsDb will be particularly attractive to communities and researchers interested in OS, and that the HOsDb will considerably facilitate research regarding the pathogenesis of OS.

## Supplementary information

**Additional file 1.**

## Data Availability

The datasets generated and analyzed during the current study are available in the HOsDb (www.hosdatabase.com).

## Abbreviations

CGH: Comparative genomic hybridization
CNV: Copy number variations
DEG: Differentially expressed gene
DEM: Differentially expressed miRNA
FDR: False discovery rate
GEO: Gene Expression Omnibus
HOsDb: human OS database
miRNA: microRNA
OS: Osteosarcoma
SNP: Single nucleotide polymorphism
TF: Transcription factor
